# A Comparative Investigation of the Bile Microbiome in Patients with Choledocholithiasis and Cholecystolithiasis through Metagenomic Analysis

**DOI:** 10.3390/ijms25063297

**Published:** 2024-03-14

**Authors:** Wonsuk Park, Joonhong Park

**Affiliations:** 1 Division of Gastroenterology, Department of Internal Medicine, Daejeon St. Mary’s Hospital, College of Medicine, The Catholic University of Korea, Daejeon 34943, Republic of Korea; mdonekr@naver.com; 2 Department of Laboratory Medicine, Jeonbuk National University Medical School and Hospital, Jeonju 54907, Republic of Korea; 3 Research Institute of Clinical Medicine of Jeonbuk National University-Biomedical Research Institute of Jeonbuk National University Hospital, Jeonju 54907, Republic of Korea

**Keywords:** metagenomic analysis, bile microbial communities, common bile duct, choledocholithiasis, gallbladder, cholelithiasis, 16S rRNA gene sequencing

## Abstract

While the precise triggers of gallstone formation remain incompletely understood, it is believed to arise from a complex interplay of genetic and environmental factors. The bile microbiome is being increasingly recognized as a possible contributor to the onset of gallstone disease. The primary objective of this study was to investigate distinctions in the microbial communities within bile specimens from patients with choledocholithiasis (common bile duct stones) and cholecystolithiasis (gallbladder stones). We employed massively parallel sequencing of the 16S rRNA gene to examine the microbial communities within bile samples obtained from 28 patients with choledocholithiasis (group DS) and cholecystolithiasis (group GS). The taxonomic composition of the bile microbial communities displayed significant disparities between the group DS and the group GS. Within the 16 prevalent genera, only *Streptococcus*, *Ralstonia*, *Lactobacillus*, and *Enterococcus* were predominantly found in the group GS. In contrast, the group DS displayed a more diverse range of genera. The alpha diversity of bile specimens was also notably lower in the group GS compared to the group DS (*p* = 0.041). Principal coordinate analysis unveiled distinct clustering of bile microbial communities depending on the location of the gallstone. Linear discriminant analysis effect size analysis, with a score threshold of >3 and the Kruskall–Wallis test (α < 0.05), recognized *Bacilli* and *Lactobacillales* as potential taxonomic markers for distinguishing patients with cholecystolithiasis limited to the gallbladder. Significant variations were found in the distribution and diversity of bile microbial communities between patients with choledocholithiasis and cholecystolithiasis. This observation suggests that alterations in the bile microbiome may contribute to the development of gallstones in these patients.

## 1. Introduction

Gallstone disease is a prevalent medical condition impacting millions of individuals across the globe. Gallstones are solid deposits that can develop in the gallbladder or bile duct, composed of a mixture of various substances such as calcium bilirubinate, calcium carbonate, calcium palmitate, calcium phosphate, glycoprotein, fatty acids, and cholesterol [1]. The formation of gallstones is an intricate process influenced by a range of factors, including genetic and environmental elements. Additionally, the bile microbiome, the collection of microorganisms residing in the bile, has been increasingly acknowledged as a potential contributor to gallstone disease development [2,3]. The gut–biliary microbiome, which encompasses the microbial communities in the gut and bile, plays a crucial role in all phases of gallstone formation. The gut microbiota can impact bile acid metabolism and absorption, thereby influencing bile composition and properties, potentially leading to gallstone formation [4,5]. The biliary microbiome itself can also contribute to gallstone development by facilitating the precipitation and accumulation of cholesterol and other biliary components. Research has revealed distinct differences in the gut–biliary microbiome between individuals with gallstone disease and those without. Patients with gallstone disease tend to have a less diverse bile microbiome, with a higher prevalence of certain bacterial genera, such as *Enterobacteriaceae*, including *Klebsiella* and *Escherichia*. This has been demonstrated using both molecular analysis [6] and cultivation techniques [7,8]. The microbial communities in the bile duct resemble those in the duodenum more than in other gastrointestinal regions, suggesting the duodenal microbiome’s significance. However, the diversity of the biliary microbiota is lower compared to the duodenal microbiota [9]. Another pertinent concern is the origin of bile microbial communities. It is suspected that retrograde infection with intestinal bacteria from the duodenum serves as the primary source of biliary infections [10,11]. The major duodenal papilla acts as the sole anatomical barrier separating the duodenum and bile duct, appearing to be the gateway for the potential ascending invasion of intestinal bacteria [9]. Protective mechanisms, such as the sphincter of Oddi, the immunological defense system, and the antimicrobial activity of bile salts, serve as defenses against intestinal bacteria invasion [7]. Previous studies demonstrated that the microbial communities of three upper gastrointestinal (GI) tract sites (saliva, stomach, and duodenum) shared similarities in bacterial types. Given the proximity of the upper GI tract to the biliary tract, it is more likely to be the primary source of bile bacteria than the lower GI tract [12].

Modern sequencing technologies, like massively parallel 16S rRNA gene sequencing and shotgun metagenomics, have facilitated more comprehensive examinations of the bile microbiome. These techniques offer detailed insight into the taxonomic composition and functional capabilities of the biliary microbiome, including the identification of previously uncultured or unknown bacterial species. Massively parallel sequencing (MPS) analysis can also unveil changes in the biliary microbiome associated with specific health conditions like gallstones or inflammation, potentially revealing valuable biomarkers or therapeutic targets. Thus, MPS has significantly broadened our knowledge of the biliary tract microbiome and its role in health and illness [6,9,13]. Various studies have employed metagenomic sequencing to illustrate variations in the metabolic profile, bacterial diversity, and physiological states of the microbial communities in the CBD for choledocholithiasis [6,14,15] and the gallbladder for cholecystolithiasis [16,17]. However, as far as we are aware, no research has compared metagenomic differences in bile microbial communities based on the gallstone’s location in patients with choledocholithiasis or cholecystolithiasis. Such a comparative investigation is essential to assess the potential impact of the microenvironment on biliary bacteria between the gallbladder and bile duct and determine the extent of bacterial compositional changes in the less hospitable biliary system.

This study aimed to explore the distinctions in microbial communities within the bile of patients affected by these two gallstone-related ailments and to assess whether any substantial disparities existed between the two groups. By scrutinizing the bile microbiome in individuals with choledocholithiasis and cholelithiasis, this research may contribute to a deeper understanding of the microbiome’s role in these medical conditions.

## 2. Results

The study comprised 28 patients, of whom 11 (39%) were male (Table 1). The median age of the entire patient cohort was 62 years, with an age range spanning from 27 to 88 years. Within this patient population, ten individuals with choledocholithiasis (group DS) presented gallstones exclusively in the CBD, while seven had gallstones in both the CBD and the gallbladder. In contrast, all 11 individuals with cholecystolithiasis (group GS) exclusively had gallstones within the gallbladder. Notably, there were no significant differences in the size, number, or radiopacity of the gallstones between the two groups. However, it is important to mention that gallstone recurrence was only observed in the group DS. Additional baseline characteristics for both the DS and group GS are shown in Table 1.

### 2.1. Taxonomic Composition Proportions in Bile Microbial Communities

**Figure 1:**
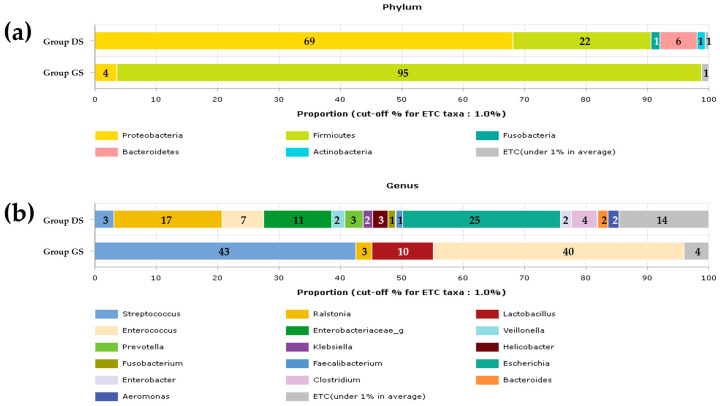
Representation of the averaged taxonomic composition proportions within the bile microbial communities at the phylum and genus levels for the group DS and the group GS. Stacked bar charts illustrating the taxonomic composition at the phylum (**a**) and genus (**b**) levels. ETC (et cetera) refers to the population of identified phylum or genus strains less than 1%. The taxonomic profiling of the microbiome at the genus level reveals that 1% of the composition in higher taxonomic ranks is classified as unassigned in group DS, whereas none is observed in group GS. At the phylum level, unassigned taxa were not identified in either group DS or GS. The x-axis represents the value in percentage.

Five dominant phyla and 16 prevalent genera were identified within the microbial communities in both the DS and group GS. Notably, there were significant variations in the relative abundance and prevalence of these bacterial taxa between the two groups. The dominant phyla in the group DS included Proteobacteria, Firmicutes, Fusobacteria, Bacteroidetes, and Actinobacteria. In contrast, the group GS was primarily dominated by *Proteobacteria* and *Firmicutes* (Figure 1a). Within the 16 prevalent genera, only *Streptococcus*, *Ralstonia*, *Lactobacillus*, and *Enterococcus* were predominantly found in the group GS. In contrast, the group DS displayed a more diverse range of genera. (Figure 1b). The proportion of Proteobacteria was notably higher in the group DS compared to the group GS, with significant differences observed in the composition of *Proteobacteria*, particularly the presence of *Ralstonia*. In contrast, *Firmicutes*, including *Enterococcus*, *Lactobacillus*, and *Streptococcus*, were more abundant in the group GS than in the group DS. However, the composition of Firmicutes itself did not significantly differ between the two groups. Additionally, *Verrucomicrobia* was exclusively found in the group DS.

**Figure 2:**
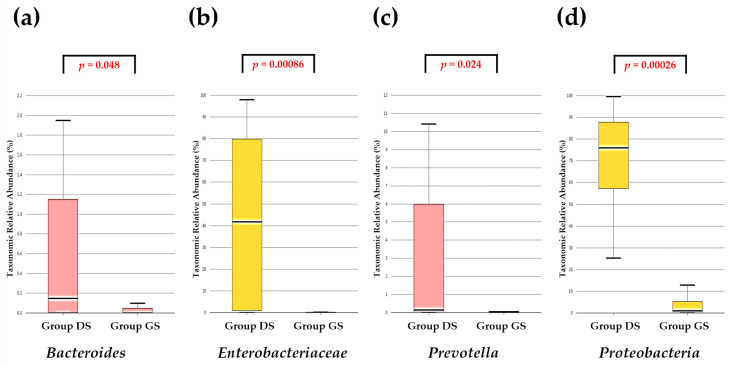
Comparative analysis of taxonomic composition within four selected taxa known for their significance in the human gastrointestinal tract. The group DS exhibited a higher relative taxonomic abundance of *Bacteroides* (**a**), *Enterobacteriaceae* (**b**), *Prevotella* (**c**), and *Proteobacteria* (**d**) compared to the group GS.

We also conducted a comparative analysis of the taxonomic composition within four selected taxa—*Bacteroides*, *Enterobacteriaceae*, *Prevotella*, and *Proteobacteria*—known for their significance in human gut microflora. Significant differences in the relative abundance of these taxa were observed between the DS and group GS (Figure 2).

### 2.2. Alpha Diversity Analysis in Bile Microbial Communities

**Figure 3:**
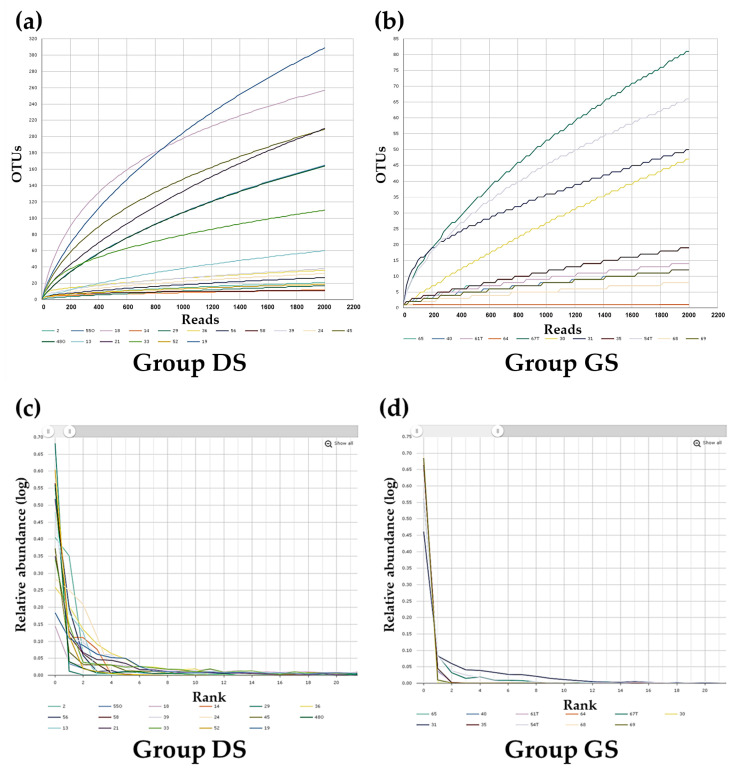
Visualization of rarefaction and rank abundance curves for the DS and group GS. Rarefaction curves and species richness indices indicate the extent of comprehensive sampling in group DS (**a**) and group GS (**b**). The broader span of rank abundance curves reflects higher relative species abundance, and the smoother curve on the Y-axis signifies greater evenness in the bacterial distribution in group DS (**c**) and group GS (**d**).

To assess species richness and diversity in both the group DS and the group GS, we employed rarefaction curves based on the number of sequences obtained for each sample. In this study, the rarefaction curves in both group DS (Figure 3a) and group GS (Figure 3b) suggest that the sequencing depth was adequate for estimating species richness and diversity within each sample. Rank–abundance curves were used to compare the microbial community structures of bile samples in group DS (Figure 3c) and group GS (Figure 3d).

**Figure 4:**
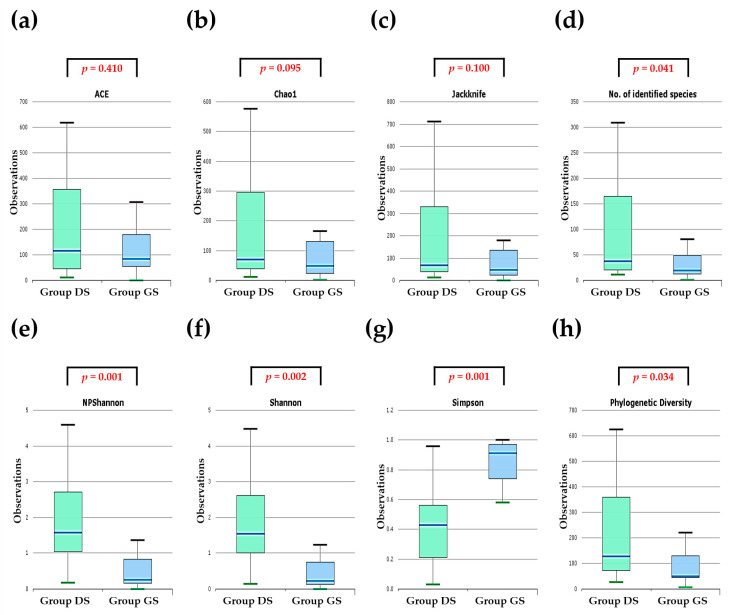
Analysis of alpha diversity in bile microbial communities in the DS and group GS at the genus level using massively parallel 16S rRNA gene sequencing. Evaluation of alpha diversity for species richness (**a**–**d**) and diversity index (**e**–**h**) within bile microbial communities collected from the common bile duct in the choledocholithiasis (group DS) and the gall bladder in the cholelithiasis (group GS).

The differences observed in the shapes of the rank–abundance curves suggest distinct microbial community structures between the two groups. An in-depth analysis of alpha diversity using diversity indices, such as ACE, Chao1, and Jackknife, was performed to explore the variations in bile microbial communities between the groups. The results of these indices indicate that bile specimens from the gallbladder in the GS group showed no significant difference in bacterial diversity compared to those from the CBD because this difference was not statistically significant (Figure 4a–c). However, when we normalized the reads of these specimens to a uniform number based on gene copy numbers, the alpha diversity, as measured by the number of identified species for species richness, was significantly lower in the group GS compared to the group DS (Wilcoxon rank sum test, group DS vs. group GS, *p* = 0.041) (Figure 4d). Additionally, alpha diversity analysis using NPShannon (*p* = 0.001), Shannon (*p* = 0.002), Simpson (*p* = 0.001), and phylogenetic (*p* = 0.034) diversity indices demonstrated statistically significant differences between the groups (Figure 4e–h).

### 2.3. Beta Diversity Analysis in Bile Microbial Communities

**Figure 5:**
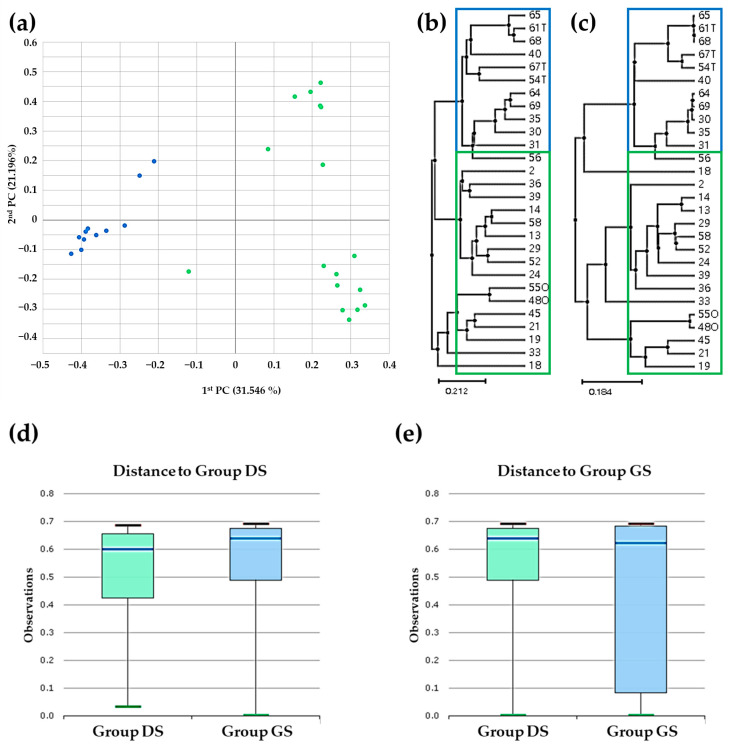
Beta diversity assessment within bile microbial communities in the DS and group GS at the genus level using massively parallel 16S rRNA gene sequencing. (**a**) Principal coordinate analysis (PCoA) highlighting distinct clustering, indicative of differences in overall bile microbial communities between the group DS (blue solid dot) and the group GS (green solid dot) based on gallstone location. (**b**,**c**) Hierarchical clustering analysis using the unweighted pair group method with arithmetic mean (UPGMA) revealed variations in abundance and diversity between the group DS (blue empty box) and the group GS (green empty box) based on generalized UniFrac (**b**) and UniFrac (**c**). (**d**,**e**) Calculation of diversity index differences between the group DS and the group GS presented in a representative box plot using permutational multivariate analysis of variance (PERMANOVA).

We performed beta diversity analysis of the bile microbial communities within the groups at the genus level utilizing massively parallel 16S rRNA gene sequencing. Our analysis involved principal coordinate analysis (PCoA) employing generalized UniFrac and UniFrac metrics. The outcomes demonstrated distinct clustering patterns between the group DS (represented by blue solid dots) and the group GS (represented by green solid dots) based on the gallstone location (Figure 5a). We also utilized the unweighted pair group method with arithmetic mean (UPGMA) hierarchical clustering analysis to reveal differences in abundance and diversity between the group DS (blue empty boxes) and the group GS (green empty boxes) using generalized UniFrac (Figure 5b) and UniFrac (Figure 5c). We calculated and presented diversity indices in a representative box plot using permutational multivariate analysis of variance (PERMANOVA) to quantitatively assess the diversity differences between the two groups (Figure 5d,e). The results highlighted significant dissimilarities in the bile microbial communities between patients with and without gallstones, suggesting a potential role for these communities in the formation of gallstones (*p* = 0.008).

### 2.4. Discovery of Taxonomic Biomarkers in Bile Microbial Communities

**Figure 6:**
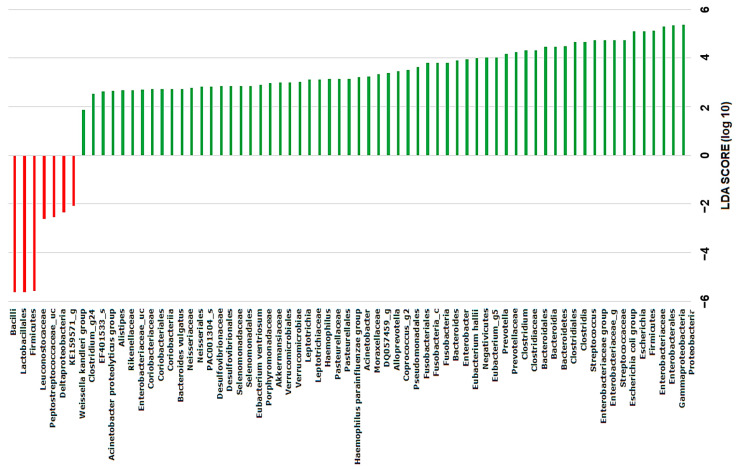
Illustration of the taxonomic distribution in the DS and group GS generated by linear discriminant analysis effect size (LEfSe) with an LDA score threshold > 3 and the Kruskall–Wallis test set at a significance level of 0.05.

We utilized linear discriminant analysis effect size (LEfSe) analysis to pinpoint taxonomic biomarkers that exhibited significant differences between the group DS (comprising patients with gallstones in the CBD and the group GS (comprising patients with gallstones in the gallbladder). We identified six genera as potential biomarkers, including *Bacilli* and *Lactobacillales*, which displayed the most substantial distinctions between the two groups. In our analysis, we employed a linear discriminant analysis (LDA) score threshold of >3, along with the Kruskall–Wallis test utilizing a significance level of α < 0.05. The findings indicate that these specific taxa have the potential to serve as biomarkers for distinguishing patients with different types of gallstones (Figure 6).

## 3. Discussion

The inquiry into the contribution of bacteria to gallstone formation is a longstanding question. With the advent of omics technologies, bacterial genes associated with gallstones have been unequivocally identified. In this study, we employed massively parallel 16S rRNA gene sequencing to explore the differences in the metagenomic profiles of microbial communities in bile, focusing on the location of gallstones. Specifically, we compared microbial communities in bile from the CBD in group DS and from the gallbladder in group GS. Our investigation confirmed the presence of several bacterial phyla in bile, including Proteobacteria, Firmicutes, Fusobacteria, Bacteroidetes, and Actinobacteria. These findings are consistent with prior research, which identified these phyla as among the most abundant in bile. Of note, in contrast to some earlier studies, our analysis did not detect the phylum *Synergistetes* in the samples [6,14,16]. The bile microbial communities in group DS exhibited higher bacterial diversity and shared more similarities with intestinal microbiota than those in group GS. This observation suggests that the environment within the CBD of choledocholithiasis patients resembles the normal digestive tract. Previous studies indicated a correlation between gut microbiota and increased inflammation, as well as the development of gallstones [18,19]. Additionally, research has highlighted connections between the microbiota in the CBD and conditions like primary sclerosing cholangitis [20], gallstones [6], and upper GI tract [9]. Our study found that the microbial composition in the bile of choledocholithiasis patients was more intricate than that of cholecystolithiasis patients and resembled typical intestinal microbiota. Genera like *Prevotella, Bacteroides*, and *Bifidobacterium* may serve as reliable indicators for assessing dietary habits and lifestyle [21]. For example, *Prevotella* has been linked to non-industrial, agrarian societies with diets primarily based on vegetables rich in polysaccharides and fiber [22]. In contrast, *Firmicutes* (specifically the *Enterococcus* genus) and *Proteobacteria* (particularly the *Enterobacteriaceae* family) were commonly found in the bile of gallstone patients [23].

The microbial communities in the bile of individuals between group DS and group GS exhibited substantial differences in terms of the types and quantities of specific bacterial strains. Prior research indicated that individuals with choledocholithiasis tend to have higher levels of *Firmicutes* and *Proteobacteria* in their bile, particularly *Enterobacteriaceae* [24]. Factors like bacterial slime, bacterial resistance in bile, and the formation of biofilms are presumed to play crucial roles in gallstone formation. Notably, prolonged exposure to bile salts is known to induce biofilm formation among enteric pathogens within the Enterobacteriaceae family. This pertains to extensively studied bacteria, such as *Salmonella* and *Shigella* species, as well as emerging pathogens, including *E. coli*, *K. pneumoniae*, *Enterococcus* spp., and *Clostridium* spp. [25]. Furthermore, Biofilm formation and anaerobic energy metabolism are considered potential microbial mechanisms involved in gallstone formation. The bacterial composition of stones and identified enterobacteria such as *Enterobacter* spp., *Enterococcus* spp., *Escherichia* spp., *Klebsiella* spp., and *Salmonella* spp. as contributors to gallstone formation. In our investigation, we observed a reduced diversity of bacteria in the bile samples from individuals in group GS compared to those from individuals in group DS. This decreased bacterial diversity in the bile of cholecystolithiasis patients may be attributed to the stagnant bile conditions that are often associated with this condition. Stagnant bile can foster the overgrowth of particular bacterial species, such as *Enterococcus* spp., which can contribute to the development of gallstones. Additionally, the group GS exhibited significantly lower alpha diversity, as determined by species richness, compared to the group DS.

The microbial composition in the bile of patients with gallstones determined by 16S rRNA amplicon sequencing displayed lower diversity compared to the microbiota found in the duodenum [9]. While many bacterial taxa were reduced in bile samples, there were notable abundances of the *Enterobacteriaceae* genera, such as *Klebsiella*, *Escherichia*, and *Pyramidobacter* [26]. The connection between decreased microbial diversity and gallstone disease has been emphasized by previous research [9,16,27,28], highlighting the need to consider an individual’s overall gut microbiota composition when assessing their risk of developing this condition. Patients with recurrent cholelithiasis may exhibit an imbalance in their bile microbial communities, potentially contributing to gallstone formation [15]. Bile microbial communities in patients with primary CBD stones appear to be more evenly distributed than those in patients with recurrent CBD stones. Among patients with recurrent CBD stones, *Proteobacteria* and *Firmicutes* are the predominant genera, with high levels of *Proteobacteria* and *Synergistetes* and lower levels of *Bacteroidetes* and *Actinobacteria*. In this study, *Bacilli* and *Lactobacillales* could serve as potential biomarkers for distinguishing patients with cholelithiasis in the gallbladder, as identified by LEfSe analysis with an LDA score threshold of >3 and the Kruskall–Wallis test (α < 0.05). Metabolic profiling of bile microbial communities in a prior study [6] demonstrated that bile samples were enriched in pathways related to glutathione reductase and putative iron-dependent peroxidase, which are associated with oxidative stress resistance. This implies that bile microbial communities play a role in maintaining redox metabolism and bacterial balance. The observed increase in flagellar assembly suggests that the microbes in the biliary environment may be more mobile. Bile specimens displayed enrichment in pathways related to ascorbate/aldarate metabolism, propanoate metabolism, and glycolysis/gluconeogenesis, whereas starch/sucrose and pentose phosphate metabolism pathways were depleted [29].

This study had several limitations. Firstly, both groups of patients underwent invasive procedures, such as endoscopic retrograde cholangiopancreatography (ERCP) or laparoscopic cholecystectomy (LC), which makes it challenging to distinguish the impact of the procedure itself from the effects of the underlying disease. Secondly, patients with choledocholithiasis or cholelithiasis had complex medical histories, including conditions like diabetes, obesity, and high cholesterol, which could have influenced the composition of their bile microbial communities. Thirdly, while we excluded patients who had used antibiotics within the past three months, some of them may have taken other types of medications during this period, introducing potential bias into the results. Future studies should collect more comprehensive information on medication usage in the months leading up to ERCP to mitigate this potential bias. Fourthly, it is important to acknowledge that the study had a relatively small sample size, which may constrain the ability to detect smaller effects and generalize the findings to a broader population. However, it is essential to consider the challenges associated with obtaining bile samples from healthy individuals. Ethical and safety considerations are paramount, given that collecting bile samples usually involves invasive procedures such as ERCP, percutaneous transhepatic cholangiography, or surgery. These procedures inherently carry risks, including infection, bleeding, and pancreatitis. Subjecting healthy individuals to such risks for research purposes raises ethical concerns that must be carefully addressed. Even though LEfSe is a valuable tool for differential abundance analysis and can help mitigate spurious bias related to a small sample size, it still has limitations due to the sample size. This suggests that there may be some aspects that were not explored in this study and that further investigations with larger sample sizes would be advantageous.

## 4. Materials and Methods

### 4.1. Specimen Collection

A total of 28 patients who had been diagnosed with either choledocholithiasis or cholecystolithiasis were enrolled at the Department of Internal Medicine, Daejeon St. Mary’s Hospital (Daejeon, Republic of Korea). The first group (DS; n = 17) consisted of patients with choledocholithiasis who were treated by ERCP. The second group (GS; n = 11) consisted of patients with cholecystolithiasis who were treated by LC. The inclusion criteria for patient selection in the study were stringent, requiring individuals to meet specific requirements. These criteria included having no history of endoscopic sphincterotomy or biliary surgery, the absence of acute cholangitis or acute cholecystitis at the time of diagnosis, no antibiotic therapy for three months preceding procedures, and not taking probiotics or any other medications known to significantly impact the gut microbiome. This was performed to ensure that the study results were not confounded by previous treatments or acute illnesses that could impact the microbial communities in the bile. A 10 mL bile specimen was collected from each patient during either ERCP or LC, and the collection procedure was performed in a sterile manner. During ERCP, bile specimens were collected using side-viewing endoscopes (TJF240/JF-260V; Olympus, Tokyo, Japan) and sterile sphincterotome catheters to avoid contamination. During LC, bile specimens were aspirated from the gallbladder into sterile disposable syringes before removing the gallbladder. All specimens were immediately placed in sterile Falcon 15 mL conical tubes (Corning Inc., New York, NY, USA) and stored at −80 °C until further analysis. This procedure ensured that the bile specimens remained in a stable condition until the bile microbiome was analyzed.

### 4.2. DNA Extraction

Total DNA was extracted from each non-centrifuged bile specimen using the FastDNA^®^ SPIN Kit for Soil (MP Biomedicals, Santa Ana, CA, USA) following the manufacturer’s protocol. The quantity of DNA was measured using a Qubit 2.0 Fluorometer (Life Technologies, Carlsbad, CA, USA), and the quality of DNA was estimated using the E-Gel electrophoresis system (Life Technologies).

### 4.3. Massively Parallel 16S rRNA Gene Sequencing

Polymerase chain reaction (PCR) amplification was performed with the extracted DNA using fusion primers that targeted the V3 to V4 regions of the 16S rRNA gene. These fusion primers were designed for bacterial identification and were as follows: 341F (5′-AATGATACGGCGACCACCGAGATCTACACXXXXXXXXCGTCGGCAGCGTCAGATG TGTATAAGAGACAGCCTACGGGNGGCWGCAG-3′) and 805R (5′-CAAGCAGAAGACGGCATACGAGATXXXXXXXXGTCTCGTGGGCTCGGAGATGTGTATAAGAGAC AGGACTACHVGGGTATCTAATCC-3′). The target region primer sequences were underlined. The fusion primers were constructed in the following order: P5 (P7) graft binding, the i5 (i7) index, nextera consensus, the sequencing adaptor, and the target region sequence. The PCR amplifications followed these conditions: initial denaturation at 95 °C for 3 min, followed by 25 cycles of denaturation at 95 °C for 30 s, primer annealing at 55 °C for 30 s, and extension at 72 °C for 30 s, with a final elongation at 72 °C for 5 min. The amplified PCR products underwent purification, and non-target products were removed using CleanPCR (CleanNA Alphen aan den Rijn, Waddinxveen, The Netherlands). The purified PCR product was assessed using 1% agarose gel electrophoresis and the Bioanalyzer 2100 (Agilent, Palo Alto, CA, USA) with a DNA 7500 chip. Mixed amplicons were pooled and subjected to 2 × 250 bp paired-end sequencing covering the amplified 16S V3-V4 region using the MiSeq Sequencing system (Illumina Inc., San Diego, CA, USA) at Chunlab, Inc. (Seoul, Republic of Korea), following the manufacturer’s instructions.

### 4.4. Bioinformatic Analysis

The raw reads were initially subjected to quality checks, and low-quality reads (Phred quality score < Q25) were filtered using Trimmomatic version 0.32. After quality control, the paired-end sequence data (mean, 35,750 reads; range, 20,239 to 72,371) were merged with the fastq_mergepairs command of VSEARCH version 2.13.4 with the default parameters [30]. Primer sequences were trimmed using Myers and Miller’s alignment algorithm with a similarity cutoff of 0.8. Non-specific amplicons that did not encode 16S rRNA were detected using nhmmer in the HMMER software package version 3.2.1 with HMM profiles [31]. Unique reads were extracted, and redundant reads were clustered using the derep_fulllength command of VSEARCH [30].

### 4.5. Analysis of Taxonomic Profiling

The 16S-based microbial taxonomic profiling (MTP) platform of EzBioCloud Apps (ChunLab, Inc., Seoul, Republic of Korea, https://www.ezbiocloud.net/; accessed on 2 February 2022) was utilized to estimate the metagenomic differences in bile microbial communities. Briefly, taxonomic assignments were performed using the EzBioCloud 16S rRNA database [32], followed by more precise pairwise alignment [30]. Chimeric reads were filtered out using the UCHIME algorithm for reads with <97% similarity [33], and the non-chimeric 16S rRNA database from EzBioCloud was used. Sequences were clustered into operational taxonomic units (OTUs) by 97% identity, and taxonomic positions of representative sequences in each OTU cluster were assigned [34]. Following chimeric filtering, reads that could not be matched to a specific species with less than 97% similarity in the EzBioCloud 16S rRNA database were gathered, and the cluster_fast command [30] was employed to carry out de novo clustering to create additional OTUs. Subsequently, OTUs consisting of a single read were excluded from further analysis. After performing taxonomic profiling on each specimen, we employed the comparative MTP analyzer within the EzBioCloud Apps for a comparative examination of the specimens. For the purpose of comparing diversity indices among specimens, read numbers were normalized via random subsampling, and the diversity indices were computed using Mothur [35]. The computed alpha diversity indices included ACE, Chao, Jackknife, NPShannon, Shannon, Simpson, and phylogenetic diversity, in addition to rarefaction curves and rank abundance curves. An alpha significance level of 0.05, along with an effect size threshold of 3, was employed as criteria for this study. Beta diversity distances were calculated to assess variations in species complexity using various algorithms, such as Bray–Curtis, Fast UniFrac, Generalized UniFrac, and Jensen–Shannon. PCoA clustering analysis was conducted using the comparative MTP analyzer to evaluate differences in species complexity. PCoA plots were generated to facilitate a comparison of microbiota composition among specimens [36]. LEfSe analysis was employed to identify significantly differential taxa between groups based on functional profiles predicted by the PICRUSt [37] and MinPath [26] algorithms. LEfSe places importance on both statistical significance and biological relevance in the identification of biomarkers [38].

### 4.6. Statistical Analysis

The metagenomic disparity in bile microbial communities between the two groups was assessed using statistical tests, including the Kruskall–Wallis test, Mann–Whitney U test, and Wilcoxon rank–sum test in the R software, specifically version R.3.1.2 from the R Foundation for Statistical Computing in Vienna, Austria. A significance level of *p* < 0.05 was applied to all statistical analyses.

## 5. Conclusions

Our study revealed that the bile microbial community in patients with choledocholithiasis exhibited higher diversity and increased abundance of specific bacterial strains compared to patients with cholelithiasis. The heightened diversity in the bile microbial community of choledocholithiasis patients suggests the presence of a more intricate and dynamic ecosystem within their bile. Notably, the increased prevalence of certain bacterial strains, namely *Ralstonia*, *Lactobacillus*, and *Enterococcus*, is of particular interest. These strains are known to be associated with inflammation, and inflammation is a recognized risk factor for gallstone formation. Therefore, it is plausible that these bacteria could contribute to gallstone formation by promoting inflammation within the bile ducts. Our study represents a valuable addition to the field of gallstone disease research and holds potential in the identification and development of novel and more effective strategies for the prevention and treatment of this condition. Further experiments are warranted, including the joint analysis of serum or urine metabolomics along with bile microbiota.

## Figures and Tables

**Table 1:** Baseline characteristics of patients with choledocholithiasis (group DS) and with cholelithiasis (group GS).

	Group DS (n = 17)	Group GS (n = 11)	*p* Value
Males/Female, n	8/9	3/8	0.435
Age, median year (range)	67 (27–88)	59 (30–81)	0.456
Location of gallstone, n (%)			<0.000
Common bile duct	10 (59%)	0	
Common bile duct and gall bladder	7 (41%)	0	
Gall bladder	0	11 (100%)	
Size of gallstone, n			0.419
Sludge	5 (29%)	1 (9%)	
<1 cm	6 (35%)	9 (82%)	
≥1 cm	6 (35%)	1 (9%)	
Number of gallstones, n			0.635
Single	7 (41%)	8 (73%)	
Multiple (≥2)	4 (24%)	2 (18%)	
Not available	6 (35%)	1 (9%)	
Radiopacity of gallstone, n	3 (18%)	2 (18%)	1.000
Cholesterol gallstone, n	14 (82%)	8 (73%)	0.653
Gallstone recurrence of, n	4 (24%)	0	0.132

## Data Availability

Data are contained within the article. Access to raw data is available only upon request, and this restriction is in place for non-commercial reasons.
